# The Public Health and Air Pollution in Asia (PAPA) Project: Estimating the Mortality Effects of Particulate Matter in Bangkok, Thailand

**DOI:** 10.1289/ehp.10849

**Published:** 2008-07-09

**Authors:** Nuntavarn Vichit-Vadakan, Nitaya Vajanapoom, Bart Ostro

**Affiliations:** 1 Faculty of Public Health, Thammasat University, Rangsit Campus, Klongluang, Pathumthani, Thailand; 2 Office of Environmental Health Hazard Assessment, California Environmental Protection Agency, Oakland, California, USA

**Keywords:** air pollution, Bangkok, mortality, PM_10_, time series

## Abstract

**Background:**

Air pollution data in Bangkok, Thailand, indicate that levels of particulate matter with aerodynamic diameter ≤10 μm (PM_10_) are significantly higher than in most cities in North America and Western Europe, where the health effects of PM_10_ are well documented. However, the pollution mix, seasonality, and demographics are different from those in developed Western countries. It is important, therefore, to determine whether the large metropolitan area of Bangkok is subject to similar effects of PM_10_.

**Objectives:**

This study was designed to investigate the mortality risk from air pollution in Bangkok, Thailand.

**Methods:**

The study period extended from 1999 to 2003, for which the Ministry of Public Health provided the mortality data. Measures of air pollution were derived from air monitoring stations, and information on temperature and relative humidity was obtained from the weather station in central Bangkok. The statistical analysis followed the common protocol for the multicity PAPA (Public Health and Air Pollution Project in Asia) project in using a natural cubic spline model with smooths of time and weather.

**Results:**

The excess risk for non-accidental mortality was 1.3% [95% confidence interval (CI), 0.8–1.7] per 10 μg/m^3^ of PM_10_, with higher excess risks for cardiovascular and above age 65 mortality of 1.9% (95% CI, 0.8–3.0) and 1.5% (95% CI, 0.9–2.1), respectively. In addition, the effects from PM_10_ appear to be consistent in multipollutant models.

**Conclusions:**

The results suggest strong associations between several different mortality outcomes and PM_10_. In many cases, the effect estimates were higher than those typically reported in Western industrialized nations.

Compelling epidemiologic evidence indicates that current ambient levels of airborne particulate matter (PM) in North American and Western European (NAWE) cities are associated with premature mortality and a wide range of morbidity outcomes [U.S. Environmental Protection Agency (EPA) 2004; World Health Organization (WHO) 2000]. Existing air pollution monitoring information and recent exposure assessments suggest that 6 to 10 million residents of Bangkok, Thailand, are exposed to levels of particulate matter with aerodynamic diameter ≤10 μm (PM_10_) that are as high as or higher than those in NAWE cities. A recent review of Asian cities, mostly in more developed countries, suggests that PM may also be associated with both mortality and morbidity [Health Effects Institute (HEI) 2004]. However, PM chemical composition and relevant population characteristics, such as activity patterns, background health status, and other factors related to socioeconomic status, may all contribute to differential risks in developing countries such as Thailand. In addition, studies of mortality and air pollution in cities like Bangkok, which have seasonal patterns dramatically different from those of NAWE, provide an opportunity to assess the potentially confounding aspects of seasonality. Bangkok’s climate is hot and humid throughout the year, with 24-hr average temperatures almost always above 80°F. Therefore, with the lack of a cold season, the seasonal weather patterns are very different from those observed in most previous studies.

The question remains whether residents of cities in developing countries are adversely affected by the existing levels of PM_10_ and whether the impacts per unit are similar to those experienced in developed Western countries. Improvements in the mortality data collection system and air monitoring program in Bangkok provide an excellent opportunity to examine the effects of PM_10_ and several gaseous pollutants on daily mortality for the years 1997 through 2003.

## Methods

### Data

Our study period extended from 1999 through 2003. We obtained daily mortality data from the Ministry of Public Health, which currently uses the *International Classification of Diseases, 10th Revision* (ICD-10) to categorize cause of death (WHO 1992). For all ages, we abstracted those with “non-accidental” mortality (i.e., total mortality minus accidents and homicides), respiratory-specific mortality, cardiovascular-specific mortality, and mortality for some additional subcategories including ischemic heart disease, stroke, conduction disorders, respiratory mortality for those < 1 year of age, lower respiratory infection (LRI) for those < 5 years of age, chronic obstructive pulmonary disease (COPD), asthma, and senility. The latter was included as an end point because our preliminary analysis showed a relatively low number of daily deaths from cardiovascular diseases and a high number from senility. We speculated that the high apparent mortality from senility might have been the result of mislabeling the cause of death from cardiovascular diseases to senility, especially among the elderly dying outside the hospitals. We also classified nonaccidental mortality by various age groups and by sex.

In Bangkok, five ambient and seven roadside monitoring stations have been measuring hourly ambient levels of PM_10_ since 1996; ten stations measure hourly ambient nitrogen dioxide, sulfure dioxide, and nitric oxide; and eight stations measure hourly ambient ozone. Because of road traffic congestion, we used PM_10_ data from the five ambient monitoring stations to represent general population exposure. Based on the common protocol, days with < 18 hourly readings were considered missing. We calculated 24-hr averages for NO_2_, NO (using the difference between NO_x_ and NO_2_), SO_2_, and PM_10_, with the requirement that at least 75% of 1-hr values be available on that particular day. For the 8-hr average value of O_3_, at least six hourly values from 0100 to 1800 hours had to be available, because the maximum O_3_ levels always occur during daylight. We calculated the daily concentrations for each pollutant in the analysis by taking the mean of all available monitoring stations. We used only the stations that provided at least 75% completeness of the measurements over the study period.

Daily weather data are available at two locations (the airport and city center) and are highly correlated (Ostro et al. 1999). Therefore, we used data from the metropolitan weather station in the center of Bangkok, because there were no missing values. The data obtained included average daily temperature and average daily relative humidity.

### Statistical approach

To assess the short-term effects of PM_10_ on daily mortality, we followed a common protocol developed by participants in the Public Health and Air Pollution Project in Asia (PAPA project), which included research teams representing Bangkok and Hong Kong, Shanghai, and Wuhan, China. We used Poisson regression, conditional on several independent variables, to control for temporal trends and meteorologic conditions. For the basic model, we used natural cubic spline models with smoothing for time and weather, using R software (version 2.5 with mgcv 1.3–24; R Development Core Team 2007). The natural spline model is a parametric approach that fits cubic functions joined at knots, which are typically placed evenly throughout the distribution of the variable of concern, such as time. The number of knots determines the overall smoothness of the fit. We determined the “best” core model for all nonaccidental cause mortality, controlling for time trend, seasonality, temperature, relative humidity, day of week, and public holidays, before entering an air pollutant into the model. In developing the core model, all PAPA cities examined 4–6 degrees of freedom (df) per year for the smoothing of time trend and 3 df for the smoothing of same-day lag of daily mean temperature and daily mean relative humidity. Preliminary analysis indicated that models with 4 or 5 df for time had mild autocorrelation, which would bias the standard errors. In contrast, a model with 6 df for the smoothing of time and first- and second-order autocorrelation terms resulted in no remaining serial correlation. Therefore, all subsequent models used this specification, although the results were very similar to those derived from the model unadjusted for autocorrelation. Based on the agreed-upon PAPA protocol, our core model used a lag of zero and 1 day (lag01) (i.e., the average of current day’s and previous day’s values), but single-day lags up to 5 days and moving averages of up to 5 days were also examined.

We conducted several sensitivity analyses to assess the impacts of different model specifications in our results. This included models with *a*) different lags of PM_10_, *b*) various sets of degrees of freedom for time and weather, *c*) different lags of temperature and relative humidity, and *d*) penalized splines for time and weather in place of natural splines. We also fitted co-pollutant models assessing the effects of PM_10_ with adjustment for gaseous pollutants. An influenza epidemic could be a potential confounder of the associations, a possibility we assessed in the sensitivity analysis. Unfortunately, daily death counts for influenza in Bangkok were likely to be under-reported, so we defined influenza epidemic according to whether the weekly respiratory mortality count was greater than the 90th percentile of each year.

All results are presented in terms of the excess risk (ER) per 10 μg/m^3^ of PM_10_, which was calculated from the relative risk (RR) as ER = (RR –1) ×100.

## Results

### Descriptive analysis

Table 1 summarizes the daily mortality data in Bangkok from 1 January 1999 to 31 December 2003. There was an average of 95 deaths per day from nonaccidental mortality. About 8% and 14% of the total consisted of mortality from respiratory and cardiovascular diseases, respectively, and about half of the total deaths were among those ≥65 years of age. Males make up about 64% of the total mortality in Bangkok. This may be attributable simply to the higher numbers of males in the city, possibly because of employment opportunities. We observed slightly increasing trends without apparent seasonal patterns in mortality data for Bangkok, suggesting that trend and seasonality may not be the strong confounding factors for the acute effects of PM_10_ on mortality.

Table 2 provides the statistical distributions of the air pollutants and weather data used in this analysis, which were 100% complete over the study period except for PM_10_, which had 4 missing days. Mean PM_10_ was 52 μg/m^3^, with a maximum value of 169.2 μg/m^3^, higher than in most cities in NAWE. We observed a high correlation between PM_10_ and both NO_2_ (*r* = 0.78) and O_3_ (*r* = 0.59). The weather in Bangkok was generally hot and humid. The median 24-hr temperature was 29.9°C and the median daily average humidity was 73.1%.

### Analytical results

Table 3 summarizes the results of pollutant models per 10-μg/m^3^ increase in PM_10_ for various disease-specific causes of mortality as well as age- and sex-specific mortality using lag01. We observed statistically significant associations with most of the outcomes including nonaccidental and cardiovascular mortality, and we observed a positive but nonsignificant association for this lag for respiratory mortality. The ER for nonaccidental mortality was 1.3% [95% confidence interval (CI), 0.8–1.7] for a 10-μg/m^3^ increase in PM_10_, with ER for cardiovascular and respiratory mortality of 1.9% (95% CI, 0.8–3.0) and 1.0% (95% CI, –0.4 to 2.4), respectively. With respect to subclassifications of cardiovascular disease, many were associated with PM_10_, with mortality from stroke demonstrating a particularly elevated risk. Among the subgroups of respiratory mortality, we observed elevated excess risks for young children, especially among infants with respiratory causes, and asthma. Some of these estimates had very wide CIs, likely due to the small number of mortality counts for these outcomes. As indicated above, we also examined death from senility and found an excess risk of 1.8% (95% CI, 0.7–2.8) which was similar to that of cardiovascular at ≥ 65 years of age.

Analysis of nonaccidental mortality by age group indicated that the effects of PM_10_ increased with age, with the strongest effects for ages ≥ 75 years. However, associations were observed for all of the other age groups and, as indicated above, for respiratory mortality for children < 1 year of age. Our analysis by sex demonstrated relatively similar effects for males and females.

Table 4 summarizes the effects of different lags of PM_10_ on several mortality outcomes. For nonaccidental and ≥ 65 mortality, of the single-day lags, unlagged PM_10_ provided the highest ER. For cardiovascular and respiratory mortality, the highest ER was observed for single-day lags of 1 and 3 days, respectively. However, for all end points, cumulative averages of 5 days of pollution generated the highest risk estimates.

Table 5 summarizes the results of the sensitivity analysis, with a focus on all-cause and cardiovascular mortality. The table indicates the effects on the ER for different df in the smoothing of time, and for multipollutant models. We examined models with 3 to 15 df per year for time, and the results were generally insensitive to the number of df specified. In addition, the inclusion of SO_2_, NO_2_, or O _3_ in the model had either no effect or slightly attenuated the estimated effect of PM_10_. Finally, the results were generally insensitive to different lags and df for smoothing for temperature and humidity (however, overall, a lag0 temperature and humidity smooth term provided the best model fit, based on the percent of the explained deviation), use of penalized spline models, and inclusion of a term for influenza epidemics. In addition, the results for senility and for cardiovascular together with senility were similar and generally insensitive to the model specifications indicated above.

## Discussion

The results of our analysis of 5 years of data from Bangkok, Thailand, indicate a statistically significant association between daily mortality and daily concentrations of PM_10_. For PM_10_, the effect estimates for nonaccidental, cardiovascular, respiratory, and age ≥ 65 (nonaccidental) mortality are generally similar to (but in the high range) of those found elsewhere (U.S. EPA 2004). A 10-μg/m^3^ increase in lag01 PM_10_ was associated with an excess risk in nonaccidental, cardiovascular, respiratory, and age ≥ 65 mortality of 1.3, 1.9, 1.0, and 1.5%, respectively. These estimates are generally similar to those reported by Ostro et al. (1998, 1999) and Vajanapoom et al. (2002) in studies of Bangkok covering earlier years. However, these studies largely used PM_10_ data estimated from airport visibility rather than the direct measurements of PM_10_ used here.

Excess risks from PM_10_ were observed for many of the cardiovascular- and respiratory-disease specific subclasses of mortality, with particularly high risks related to respiratory diseases for those < 1 year of age, asthma, LRI, stroke, and senility. The similar magnitudes of the excess risks on cardiovascular age ≥ 65 years and senility suggested that the latter probably includes cardiovascular mortality that has been incorrectly classified, especially for the elderly dying outside of hospitals, where the cause of death is often diagnosed as senility by a nonphysician coroner. Analysis by age indicated associations with PM_10_ for all of the subgroups, and our examination of lags indicated that multiday averages of 5 days generated the largest effect estimates. In addition, many of the PM_10_ associations were retained in multipollutant models. The results of the sensitivity analyses indicate that our core model was generally robust to choices of model specifications, spline model used, degrees of freedom of time smoothers, lags for temperature, adjustment for autocorrelation, and adjustment for influenza epidemics.

Generally our analysis of PM_10_ per 10 μg/m^3^ in Bangkok generated effect estimates that are higher than most previously reported. For example, our estimate for nonaccidental mortality is 1.3% (95% CI, 0.8–1.7%). In comparison, an analysis of 75 single-city time-series analyses from around the world generated an estimate of 0.6% (95% CI, 0.5–0.7%) (Anderson et al. 2005). A study of the 90 largest cities in the United States gave an estimate of 0.2% (95% CI, 0.1–0.4%) (Dominici et al. 2003), whereas a study of 29 European cities yielded an estimate of 0.6% (95% CI, 0.4–0.7%) (Katsouyanni et al. 2003). A study of 14 cities in the United States using a case–crossover approach generated an estimate of 0.35% (95% CI, 0.2–0.5%) (Schwartz 2004). A meta-analysis of Asian studies using a random-effects estimate gave an estimate of 0.49% (95% CI, 0.23–0.76%) based on four cities: Bangkok; Seoul and Inchon, South Korea; and Hong Kong (HEI 2004). Thus, it is clear that the results for Bangkok are at the upper end of the range of estimates. It is also significant that some high estimates have been reported in other less-developed countries. For example, a study in Mexico City reported an excess risk of 1.8% (95% CI, 0.9–2.7%), whereas analysis of Santiago, Chile, found an excess risk of 1.1% (95% CI, 0.9–1.4%) (Castillejos et al. 2000; Ostro et al. 1996).

We can speculate on several possible reasons for our findings, including *a*) differences in particle chemistry in Bangkok; *b*) the proximity of a large proportion of the population to roads and traffic congestion; *c*) the likely high penetration rates due to low prevalence of home air conditioning in favor of open ventilation between indoors and outdoors (Tsai et al. 2000); *d*) the greater duration of exposure due to the amount of time spent outdoors, because many Thais work and eat outdoors; *e*) factors related to lower economic development and socioeconomic status, such as lower background health status and use of health care, and higher smoking rates and co-morbidity; *f* ) greater exposure to indoor sources such as incense and cooking; and *g*) stochastic variability. Because of several of these factors (although only anecdotal in nature), it is likely that the effective inhaled dose of any given concentration measured from a fixed site outdoor monitor is greater in Bangkok than in Western industrialized countries.

To date, few studies that relate mortality to air pollution have been conducted in Asia. Studies of daily mortality have been conducted in Inchon (Hong et al. 1999), Seoul, and Ulsan, South Korea (Kwon et al. 2001; Lee and Schwartz 1999; Lee et al. 1999); Shenyang, China (Xu et al. 2000); seven cities in South Korea (Lee et al. 2000); and New Delhi, India (Cropper et al. 1997). For the most part, policy makers in Asia have had to draw from studies conducted in North America and Western Europe. Although it may be reasonable to extrapolate the findings from the NAWE region to other parts of the world, our study also suggests that the per-unit effects may be higher in certain developing countries. Additional studies undertaken in developing countries in Asia and other parts of the world can validate our findings and help determine the factors that might modify the effect estimate.

Finally, our analysis demonstrated an association between air pollution and mortality in a region that would not be confounded by cold weather and associated respiratory infections. As such, it supports the likelihood of a causal association in studies in NAWE, which experience greater seasonality and colder temperatures.

## Figures and Tables

**Table 1:** Average daily mortality in Bangkok, 1 January 1999 to 31 December 2003.

Mortality	ICD-10 codes	Deaths/day ± SD
Nonaccidental (age, years)	A00–R99	95.0 ± 12.1
< 5		3.0 ± 1.8
4–44		29.0 ± 5.9
18–50		34.0 ± 6.4
45–64		27.0 ± 5.4
> 50		66.0 ± 9.9
≥65		45.0 ± 7.9
≥75		21.0 ± 5.2
Male		61.0 ± 8.9
Female		43.0 ± 7.6
Cardiovascular	I00–I99	13.0 ± 4.3
Ischemic heart diseases	I20–I25	4.0 ± 2.3
Stroke	I60–I69	5.0 ± 2.5
Conduction disorder	I44–I49	1.0 ± 0.5
Cardiovascular ≥age 65	I00–I99	6.7 ± 3.0
Respiratory	J00–J98	8.0 ± 3.1
Respiratory < age 1	J00–J98	0.1 ± 0.4
LRI < 5 years	J10–J22	1.0 ± 0.4
COPD	J40–J47	2.0 ± 1.0
Asthma	J45–J46	1.2 ± 0.4
Respiratory > age 65	J00–J98	3.5 ± 2.0
Senility	R54	14.0 ± 4.2

**Table 2:** Distribution of air pollutants and meteorology data in Bangkok, 1 January 1999 to 31 December 2003.

					Percentiles					
Pollutants and meteorology	Mean	Min	Max	SD	5th	25th	50th	75th	95th	No. of days
PM_10_ (μg/m^3^)	52.1	21.3	169.2	20.1	29.6	38.9	46.8	59.9	93.2	1,822
SO_2_ (μg/m^3^)	13.2	1.5	61.2	4.8	7.1	10.1	12.5	15.6	21.0	1,826
NO_2_ (μg/m^3^)	44.7	15.8	139.6	17.3	24.4	31.7	39.7	54.8	79.3	1,826
O_3_ (μg/m^3^)	59.4	8.2	180.6	26.4	25.3	39.1	59.4	75.3	109.8	1,826
NO (μg/m^3^)	28.0	3.7	126.9	14.2	11.4	18.1	28.0	34.9	56.0	1,826
Temperature (°C)	28.9	18.7	33.6	1.7	25.8	28.1	29.1	29.9	31.3	1,826
Relative humidity (%)	72.8	41.0	95.0	8.3	58.0	67.8	73.1	78.3	86.0	1,826

Max, maximum; Min, minimum.

**Table 3:** Percent ER in mortality (95% CI) for a 10-μg/m^3^ increase in lag01 PM_10_ a.

Mortality	%ER (95% CI)
Cause-specific	
Nonaccidental	1.3 (0.8 to 1.7)
Cardiovascular	1.9 (0.8 to 3.0)
Ischemic heart disease	1.5 (−0.4 to 3.5)
Stroke	2.3 (0.6 to 4.0)
Conduction disorders	−0.3 (−5.9 to 5.6)
Cardiovascular ≥age 65	1.8 (0.2 to 3.3)
Respiratory	1.0 (−0.4 to 2.4)
Respiratory ≤age 1	14.6 (2.9 to 27.6)
LRI < age 5	7.7 (−3.6 to 20.3)
COPD	1.3 (−1.8 to 4.4)
Asthma	7.4 (1.1 to 14.1)
Respiratory ≥age 65	1.3 (−0.8 to 3.3)
Senility	1.8 (0.7 to 2.8)
Age-specific for nonaccidental (years)	
0–4	0.2 (−2.0 to 2.4)
5–44	0.9 (0.2 to 1.7)
18–50	1.2 (0.5 to 1.9)
45–64	1.1 (0.4 to 1.9)
≥50	1.4 (0.9 to 1.9)
≥65	1.5 (0.9 to 2.1)
≥75	2.2 (1.3 to 3.0)
Sex-specific for nonaccidental	
Male	1.2 (0.7 to 1.7)
Female	1.3 (0.7 to 1.9)

a Model covariates include smooth of time with 6 df, smooth of unlagged temperature and humidity with 3 df, and day of week.

**Table 4:** Lag effects of PM_10_ for major causes of mortality [percent ER (95% CI)].

Lag days	Nonaccidental	Cardiovascular	Respiratory	Age ≥65
Lag0	1.2 (0.8 to 1.6)	1.5 (0.5 to 2.6)	1.0 (−0.3 to 2.3)	1.5 (0.9 to 2.0)
Lag1	0.9 (0.6 to 1.3)	1.7 (0.7 to 2.7)	0.8 (−0.5 to 2.0)	1.1 (0.6 to 1.7)
Lag2	0.9 (0.5 to 1.3)	1.6 (0.6 to 2.6)	1.1 (−0.1 to 2.3)	1.1 (0.6 to 1.6)
Lag3	0.8 (0.4 to 1.2)	0.8 (−0.1 to 1.8)	1.3 (0.1 to 2.6)	1.2 (0.6 to 1.7)
Lag4	0.3 (−0.1 to 0.7)	−0.1 (−1.1 to 0.9)	0.7 (−0.6 to 1.9)	0.7 (0.2 to 1.2)
0–1 mean	1.3 (0.8 to 1.7)	1.9 (0.8 to 3.0)	1.0 (−0.4 to 2.4)	1.5 (0.9 to 2.1)
0–4 mean	1.4 (0.9 to 1.9)	1.9 (0.6 to 3.2)	1.9 (1.2 to 2.6)	1.9 (1.2 to 2.6)

**Table 5:** Percent ER (95% CI) in mortality for a 10-μg/m^3^ increase in PM_10_ with alternative degrees of freedom for smoothing of time and with adjustment for gaseous pollutants.

Model specification	%ER (95% CI)
Nonaccidental (df)	
3	1.3 (0.9 to 1.8)
4	1.2 (0.8 to 1.7)
6	1.3 (0.8 to 1.7)
6, with SO_2_	1.2 (0.8 to 1.7)
6, with NO_2_	1.0 (0.2 to 1.8)
6, with O_3_	1.1 (0.6 to 1.7)
9	1.1 (0.7 to 1.6)
12	1.1 (0.6 to 1.5)
15	1.2 (0.7 to 1.6)
Cardiovascular (df)	
3	1.8 (0.8 to 2.7)
4	1.6 (0.7 to 2.6)
6	1.7 (0.7 to 2.7)
6, with SO_2_	2.0 (0.9 to 3.3)
6, with NO_2_	2.3 (0.2 to 4.3)
6, with O_3_	1.8 (0.5 to 3.2)
9	1.7 (0.6 to 2.8)
12	1.8 (0.7 to 3.0)
15	2.2 (0.9 to 3.4)
